# Supplementary figures and images for: Transcriptomic Analysis of Genes Involved in Plant Defense Response to the Cucumber Green Mottle Mosaic Virus Infection
Source: Life (Basel). 2021 Oct 10;11(10):1064. doi: 10.3390/life11101064 (PMC8541684; doi:10.3390/life11101064)

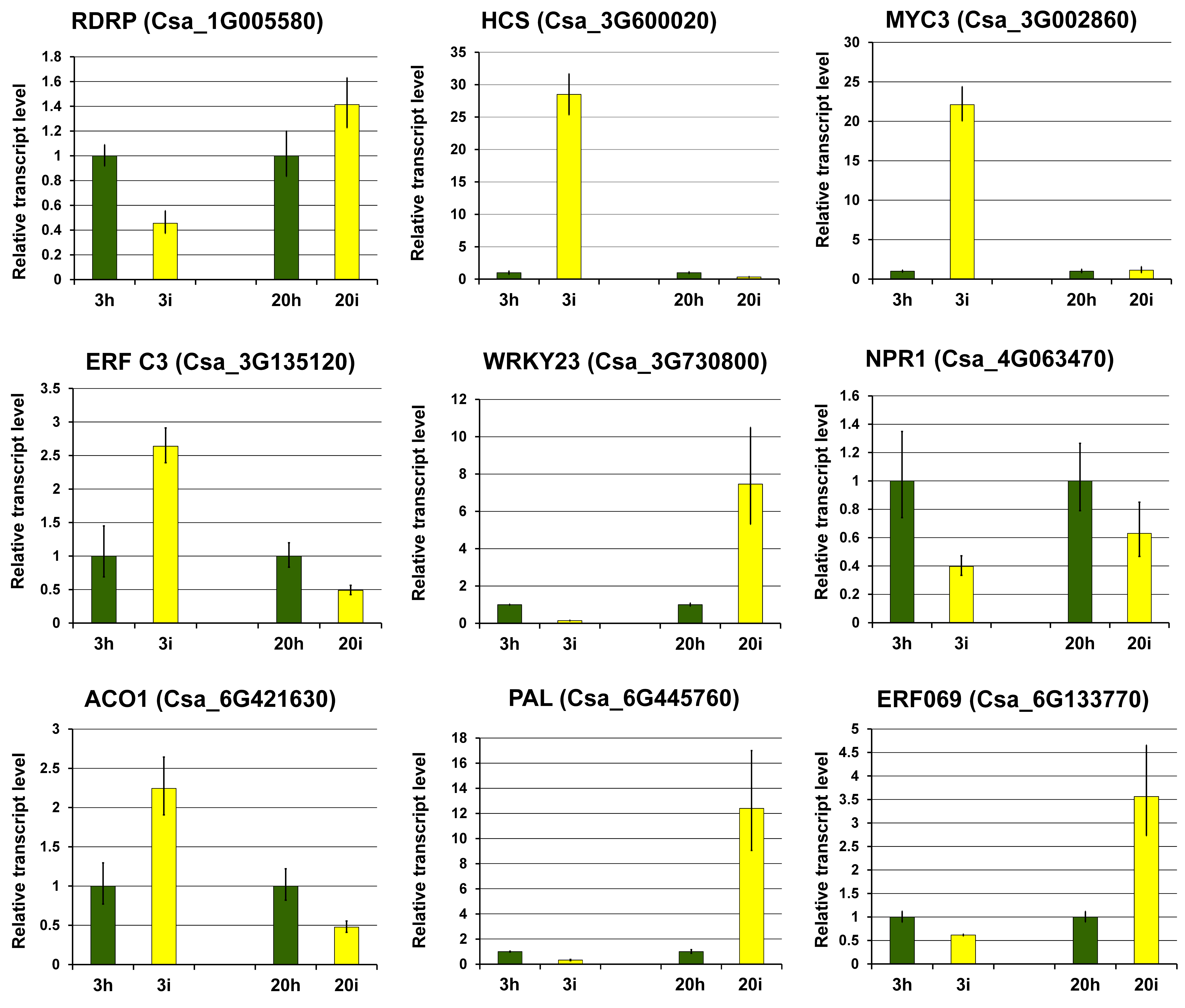

Supplement: Supplementary file 1 [file life-11-01064-s001.zip › FigureS1.tiff]
